# Controlling Freeze–Thaw Gelation of Egg Yolk via Enzymatic Treatment

**DOI:** 10.3390/gels12050430

**Published:** 2026-05-14

**Authors:** Karina Ilona Hidas, István Dalmadi, Koppány László Majzinger, Anna Visy, Adrienn Varga-Tóth, Csaba Németh, Ildikó Csilla Nyulas-Zeke

**Affiliations:** Department of Livestock Products and Food Preservation Technology, Institute of Food Science and Technology, Hungarian University of Agriculture and Life Sciences, Ménesi St. 43–45, H-1118 Budapest, Hungary; hidas.karina.ilona@uni-mate.hu (K.I.H.); majzinger.koppany.laszlo@uni-mate.hu (K.L.M.); visy.anna@uni-mate.hu (A.V.); toth.adrienn@uni-mate.hu (A.V.-T.); nemeth.csaba@capriovus.hu (C.N.); nyulasne.zeke.ildiko.csilla@uni-mate.hu (I.C.N.-Z.)

**Keywords:** egg, freeze–thaw stability, gelation, enzymatic hydrolysis, functional properties

## Abstract

Freeze–thaw cycles lead to undesirable gelation in egg yolk, which negatively affects its functional properties, restricting its application in the food industry. This study aimed to investigate whether enzymatic treatment can prevent the freeze-induced gelation of egg yolk, thereby maintaining its desirable quality attributes. Egg yolk samples were treated with an enzyme preparation (Biocatalysts Flavorpro™ 750MDP) at concentrations of 0.05, 0.3, and 0.5 *w*/*w*%, homogenized, and incubated at 40 °C for 120 min, followed by rapid cooling and freezing at −24 ± 1 °C for 60 d. Control samples without enzyme treatment were subjected to the same processing steps as the other samples. After thawing, all samples were analyzed for pH, color, rheological and thermophysical properties, turbidity and visual appearance. The results demonstrated that although enzymatic treatment and its combination with freezing significantly altered color, turbidity, rheological and thermophysical properties of egg yolk, it effectively inhibited freezing-induced gel formation, particularly at 0.3 *w*/*w*%. The parameters characterizing rheological behavior—yield stress, consistency coefficient, and flow behavior index—were preserved close to those of fresh yolk after the freeze–thaw process. These findings suggest that exopeptidase treatment is a promising approach for controlling freeze–thaw-induced gelation in egg yolk, supporting its wider use in frozen and processed egg products.

## 1. Introduction

Egg is a commonly utilized raw material in the food industry due to its high nutritional value and diverse functional attributes, [1] such as emulsifying [2], foaming [3], thickening and gelling abilities [4]. In industrial practice, processed egg products, including liquid egg, egg powder, or separated egg parts, are privileged over shell eggs because of their improved microbiological safety, ease of handling, and eligibility for large-scale applications [5,6]. However, in the case of liquid egg products, the application of preservation techniques is required because of the limited shelf life [7].

Freezing is one of the most widely used preservation methods [8]. It is economically feasible, and it effectively slows down the biochemical reactions and the microbial growth [9], thereby extending the shelf life of these perishable foods. Consequently, freezing can be applied to egg products to ensure long-term storage [10] and to balance fluctuations in supply and demand. Despite its advantages, freezing may induce undesirable physicochemical changes [11], particularly in protein-rich systems, where denaturation, aggregation, and structural rearrangements can occur [12,13].

Frozen egg products are produced from fresh eggs by removing the shells, separating the parts (albumen and yolk), pasteurizing them, and then freezing. The resulting products include frozen egg whites, egg yolks and even whole eggs [14]. Frozen egg yolks are especially valued for preserving their original flavor and color since freezing effectively decelerates the velocity of lipid oxidation and browning. Furthermore, it inhibits other undesired chemical changes [15], so these products can be stored for up to 12 months. However, freezing also triggers alterations that can adversely impact the properties of the yolk [15]. During freeze–thaw cycles, egg yolk undergoes irreversible gelation, leading to a significant increase in viscosity as a result of intermolecular cross-linking, and a paste-like structure is formed [16]. This alteration negatively affects its handling characteristics and limits its application in food formulations [17].

According to Primacella et al. [18], the mechanism behind gel formation is closely related to the complex protein system in the yolk. During protein aggregation, a balance between attractive and repulsive forces is established, resulting in the formation of a highly organized three-dimensional network capable of retaining significant amounts of water [19,20]. During freezing, ice crystal formation removes water molecules from proteins and modifies both intra- and intermolecular interactions [21]. This process leads to the concentration of solutes and promotes interactions and aggregation of low-density lipoproteins (LDLs) in the yolk plasma [22].

Various approaches have been proposed to prevent or reduce freeze-induced gelation of egg yolk. The addition of cryoprotectants such as saccharides—small molecule saccharides and modified starch—or sodium chloride (NaCl) has been shown to be effective [23,24]. Saccharides form hydrogen bonds with water via hydroxyl groups, converting free water into bound water [19], reducing water migration, and protecting protein structure [25,26]. NaCl influences freeze–thaw yolk gelation: at low concentrations (<4%), it forms an LDL–water–NaCl complex, increases freezable water, protects LDL, and inhibits protein interactions and gel formation through electrostatic shielding [27,28].

However, the use of saccharides and salts is often limited due to their impact on taste and formulation constraints. Technological approaches, including mechanical treatments, for instance, homogenization [29], rapid or cryogenic freezing, have also been investigated [30,31]. Freezing rate and the formation of fine ice crystals during the process were proven to be critical for minimizing tissue damage during thawing [32]. Rapid freezing partially prevented gelation, as increased freezing rates led to the formation of smaller ice crystals. This reduces the destructive effects of ice crystals. Nevertheless, these methods may not fully eliminate gelation or may not be feasible under all processing conditions [33]. Other additives, including hydrocolloids and protein hydrolysates [34], have also been explored, yet a universally applicable solution has not been established.

Enzymatic modification represents a promising strategy for tailoring the functional properties of protein-based food systems [35]. Proteolytic enzymes are widely applied in the food industry to modify protein structure through controlled hydrolysis, leading to reduced molecular weight, increased availability of ionizable groups, and altered exposure of the hydrophobic regions. These changes can significantly influence protein–protein and protein–water interactions, thereby affecting key functional properties such as solubility, emulsifying capacity, and rheological behavior [36]. From a freeze–thaw perspective, such structural modifications may reduce the tendency of proteins to aggregate and form continuous gel networks.

Previous studies have demonstrated that proteolytic enzymes, including papain, can inhibit gelation in egg yolk by altering protein interactions [37]. Furthermore, the application of proteases and phospholipases has been reported to influence yolk structure and aggregation phenomena by Ma et al. [23]. However, limited information is available regarding the effectiveness of specific enzyme types under conditions relevant to industrial freezing and thawing. In addition, the relationship between enzymatic modification, water state, protein denaturation, and macroscopic functional properties such as rheological and emulsifying properties has not yet been fully clarified.

Therefore, the aim of the present study was to investigate the effect of enzymatic treatment using an exopeptidase preparation on the freeze–thaw stability of egg yolks. It was hypothesized that controlled enzymatic hydrolysis modifies protein interactions and water binding in a manner that inhibits aggregation and gel network formation during freezing. To test this hypothesis, egg yolk samples treated with different enzyme concentrations were subjected to a freeze–thaw cycle and analyzed in terms of physicochemical properties (pH, color, turbidity), rheological behavior, and thermal characteristics using Differential Scanning Calorimetry. The findings of this study may contribute to the development of improved strategies for maintaining the quality and functionality of frozen egg yolk products and support their wider application in the food industry.

## 2. Results and Discussion

### 2.1. Changes in pH During Freeze–Thaw and Enzymatic Treatment

The pH values of fresh and frozen–thawed egg yolk samples are presented in Table 1.

Frozen storage significantly affected the pH of the untreated control sample, increasing from 6.49 to 6.62. This rise is consistent with the behavior of protein-rich systems, where ice formation leads to the exclusion of solutes into the unfrozen phase, resulting in increased ionic strength and altered buffering conditions [38].

In fresh samples, enzymatic treatment caused a slight decrease in pH with increasing enzyme concentration; however, these changes were not statistically significant. This tendency may be attributed to the release of ionizable groups, particularly carboxyl groups (–COOH), during peptide bond cleavage [39].

After the freeze–thaw cycle, enzyme-treated samples exhibited a decreasing trend in pH with increasing enzyme concentrations, in contrast to the untreated control. Notably, the lowest pH values were measured in samples treated with 0.30% (pH = 6.31) and 0.50% (pH = 6.32) enzyme concentrations after freezing [40]. Similar pH reductions suggest that enzymatic hydrolysis partially counteracts the alkalinization following proteolytic treatment have been reported in other protein-based systems [14].

These findings align with previous reports on pH changes during frozen storage. For instance, Luo et al. [41] observed an initial decrease followed by an increase in pH during prolonged storage of egg yolk. Tan et al. [38] reported that in protein-rich systems, pH changes during freezing are strongly influenced by solute concentration effects and salt equilibria in the unfrozen phase.

Overall, the results indicate that while freezing promotes an increase in pH, enzymatic treatment modifies this response, likely through changes in protein structure and buffering capacity, which may also influence subsequent functional properties such as gelation.

### 2.2. Emulsion Stability as Inferred from Turbidity Measurements

**Figure 1:**
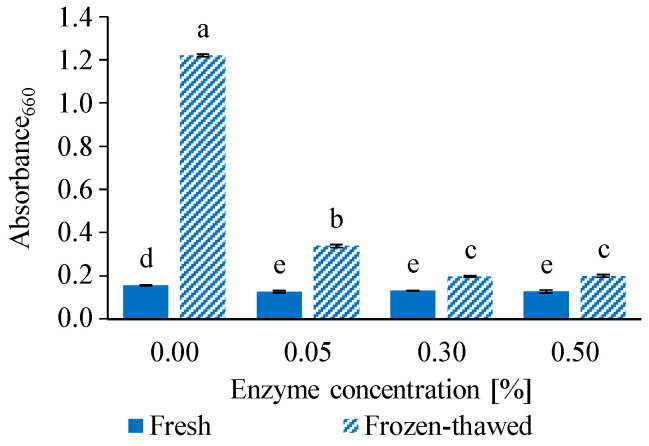
Effect of enzymatic treatment and freeze–thaw cycle on the turbidity of egg yolk samples. Turbidity was determined by measuring the absorbance at 600 nm. The results are expressed as mean ± SD, *n* = 6. ^a–e^ Different superscript letters indicate significant differences (*p* < 0.05).

The turbidity of fresh and frozen-thawed egg yolk samples is presented in Figure 1.

Frozen storage significantly increased the turbidity of the untreated control sample, indicating extensive aggregation of yolk components induced by the freeze–thaw process. This increase in optical density serves as an indicator of enhanced particle aggregation, which leads to the formation of larger, more heterogeneous structures and consequently to reduced emulsion stability [31].

In fresh samples, enzymatic treatment resulted in lower turbidity values compared to the control. This decrease can be attributed to proteolytic hydrolysis, which reduces protein size, leading to a more homogeneous dispersion with lower light scattering. Similar effects have been reported in other protein-based systems, where enzymatic treatment reduced the turbidity of pasta cooking water due to the breakdown of macromolecular structures [42].

After the freeze–thaw cycle, enzyme-treated samples exhibited significantly lower turbidity compared to the untreated control frozen sample, indicating that enzymatic modification mitigated freeze-induced aggregation. This effect was most pronounced at 0.30 and 0.50 *w*/*w*% enzyme concentrations, where the lowest turbidity values were observed.

The observed behavior suggests that enzymatic hydrolysis limits the formation of large aggregates during freezing by modifying protein–protein interactions and reducing the ability of proteins to form network structures. As a result, enzyme-treated samples retained a more stable and dispersed structure after thawing, which is indicative of improved emulsion stability [43,44].

Overall, the turbidity results demonstrate that enzymatic treatment enhances the stability of egg yolk emulsions during freeze–thaw processing. These structural changes are expected to influence the macroscopic flow behavior of egg yolk, which was further investigated by rheological measurements.

### 2.3. Color Characteristics of Egg Yolk Samples

**Figure 2:**
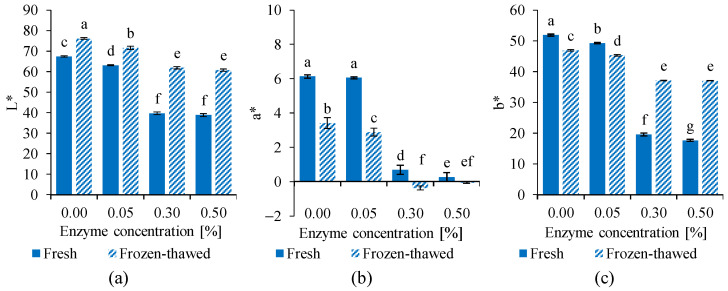
Effect of enzymatic treatment and freeze–thawing on the color parameters of egg yolk samples. Changes in CIELAB coordinates, including (**a**) lightness, L*; (**b**) redness, a* and (**c**) yellowness, b*, are presented as a function of the enzyme concentration. The results are expressed as mean ± SD, *n* = 5. ^a–g^ Different superscript letters indicate significant differences on the diagrams (*p* < 0.05).

The color parameters of fresh and frozen-thawed egg yolk samples are presented in Figure 2.

Frozen storage significantly affected the color of untreated control samples. An increase in lightness (L*) was observed, which is likely associated with the aggregation of low-density lipoprotein (LDL) particles, leading to enhanced light scattering. At the same time, a decrease in redness (a*) and yellowness (b*) was detected, indicating structural and pigment redistribution during freeze-induced gelation [45].

In fresh samples, enzymatic treatment significantly influenced the color parameters. Increasing enzyme concentration resulted in a decrease in lightness (L*), indicating a darker appearance, particularly at 0.30% and 0.50%. Simultaneously, both redness (a*) and yellowness (b*) decreased markedly, suggesting modifications in pigment–protein interactions and changes in the microstructure of the yolk.

After the freeze–thaw cycle, the combined effect of enzymatic treatment and freezing led to complex changes in the color parameters. Lightness generally increased compared to fresh samples. Yellowness (b*) decreased in the control and low enzyme concentration samples, while it increased with higher enzyme concentrations. These changes reflect structural rearrangements occurring during freezing and thawing, influenced by prior enzymatic modification [46].

The color difference (ΔE*) values between fresh and frozen–thawed egg yolk samples are presented in Table 2. Freezing induced clearly perceptible color changes in all samples, with ΔE* values exceeding the threshold of great visual differences. In addition, increasing enzyme concentration led to higher ΔE* values, indicating more pronounced color changes relative to the fresh samples. These results suggest that enzymatic treatment affects yolk color both directly and by modifying the extent of freeze–thaw-induced structural and optical changes.

Overall, the observed color changes can be attributed to modifications in protein structure affecting light scattering and pigment binding, which are closely related to the physicochemical transformations that occur during enzymatic treatment and freezing [47].

These findings are in good agreement with the turbidity results, as both parameters reflect changes in the dispersion state and aggregation behavior of the yolk components. The reduced turbidity observed in enzyme-treated samples corresponds to decreased light scattering, which is also manifested in the altered CIELAB color coordinates.

### 2.4. Rheological Properties and Flow Behavior

**Figure 3:**
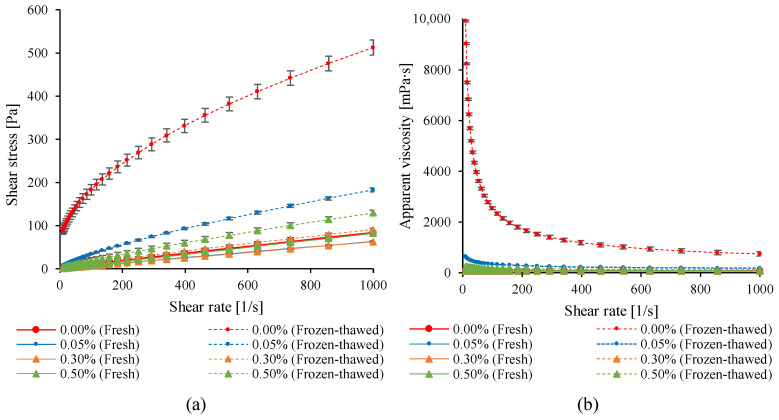
Effect of enzymatic treatment and freeze–thaw cycle on the rheological properties of egg yolk. (**a**) Flow curves and (**b**) viscosity curves. The results are expressed as mean ± SD, *n* = 3.

The rheological behavior of egg yolk samples as affected by enzymatic treatment and freeze–thaw processing is shown in Figure 3, while the parameters obtained from the Herschel–Bulkley model are summarized in Table 3.

Frozen storage had a pronounced effect on the rheological properties of the untreated control sample. After the freeze–thaw cycle, a substantial increase in shear stress and apparent viscosity was observed, particularly at low shear rates, indicating the formation of a gel-like structure. This behavior is characteristic of freeze-induced aggregation and the development of a continuous three-dimensional network [48].

In fresh samples, enzymatic treatment slightly reduced viscosity and shear stress compared to that of the control. This effect can be attributed to partial protein hydrolysis, leading to a reduced molecular size and weaker intermolecular interactions [49].

After the freeze–thaw cycle, enzyme-treated samples exhibited markedly lower shear stress and viscosity compared with those of the untreated control. This indicates that enzymatic treatment effectively mitigated the freeze-induced gelation. Among the tested concentrations, the 0.30 *w*/*w*% treatment was the most effective, as its rheological behavior closely resembled that of fresh egg yolk. These results suggest that enzymatic hydrolysis likely disrupted protein–lipoprotein interactions responsible for gel network formation [21].

All samples exhibited shear-thinning behavior, which is typical of non-Newtonian fluids. However, the extent of shear-thinning was more pronounced in the untreated frozen–thawed sample, reflecting a more rigid internal structure. In contrast, enzyme-treated samples exhibited behavior more similar to Newtonian fluids, indicating improved structural stability during freezing [50].

The Herschel–Bulkley model parameters further supported these observations (Table 3).

Freeze–thaw processing had a dramatic impact on the rheological behavior of the untreated control samples. The yield stress (τ_0_) increased from 0.00 to 51.10 Pa, accompanied by a substantial rise in the consistency coefficient (K) and a marked decrease in the flow behavior index (n). These changes indicate the formation of a highly structured, gel-like system with strong resistance to flow.

In fresh samples, enzymatic treatment reduced the consistency coefficient (K) and slightly increased the flow behavior index (n), indicating a shift toward more Newtonian-like flow behavior and a less structured system.

After the freeze–thaw cycle, enzyme-treated samples exhibited significantly lower τ_0_ and K values compared to the untreated control, demonstrating effective inhibition of gel network formation. The most pronounced effect was observed at 0.30 *w*/*w*% enzyme concentration, where the yield stress was negligible and the consistency coefficient remained relatively low. The flow behavior index (n) increased in enzyme-treated frozen–thawed samples compared to the control, indicating a less pronounced shear-thinning behavior and flow characteristics closer to those of Newtonian fluids. These results are consistent with the flow curve observations, where enzyme-treated samples exhibited reduced viscosity and weaker structural buildup.

Overall, the Herschel–Bulkley parameters confirm that enzymatic treatment limits the formation of a continuous protein network during freezing. This effect is likely related to the reduction in molecular weight and the modification of protein–protein interactions, which are essential for gel structure development.

### 2.5. Protein Denaturation Behavior Assessed by Differential Scanning Calorimetry (DSC)

The effects of enzymatic treatment and freeze–thaw processing on protein denaturation of egg yolk were evaluated by Differential Scanning Calorimetry (Table 4).

Freeze–thaw processing of the untreated control sample resulted in a pronounced decrease in denaturation enthalpy (ΔH_d_), from 1.19 to 0.84 J/g, indicating extensive structural alterations and partial denaturation of the egg yolk proteins. This confirms that freezing significantly reduces the fraction of native, thermally denaturable protein structures [51].

In fresh samples, enzymatic treatment caused a gradual decrease in ΔH_d_ with increasing enzyme concentration, indicating partial protein hydrolysis and a reduced amount of intact native structures available for thermal denaturation [52].

After the freeze–thaw cycle, no significant differences in ΔH_d_ were observed between enzyme-treated samples and the control. Although the ΔH_d_ value did not show any further significant changes after freezing, both freezing and enzyme treatment—separately and in combination—have a significant effect on native proteins.

After the freeze–thaw cycle, no significant differences in ΔH_d_ were observed between enzyme-treated samples and the control, indicating that freezing dominated the denaturation process. However, this does not imply the absence of an enzymatic effect, as differences were evident in other functional and structural properties.

The denaturation temperature (T_d_) exhibited only minor changes. In the control sample, freeze–thaw processing had little effect on T_d_, indicating that the thermal stability of the remaining structures was largely preserved.

In fresh samples, T_d_ slightly increased with increasing enzyme concentration, suggesting marginal stabilization of the residual protein structures. A similar trend was observed after freezing, where higher enzyme concentrations corresponded to slightly elevated T_d_ values. These findings suggest that the total amount of native protein decreased; however, the thermal stability of the residual structures was not adversely affected and may have been enhanced [53].

Overall, the results of DSC measurement indicate that enzymatic treatment induces partial protein modification prior to freezing, while freeze–thaw processing leads to a general reduction in the denaturable protein content. The combination of these effects confirms the hypothesis that controlled enzymatic hydrolysis may contribute to modifying protein interactions in egg yolk, which is consistent with the observed mitigation of gelation and improved functional stability after freezing.

### 2.6. Water State and Melting Properties Determined by DSC

**Figure 4:**
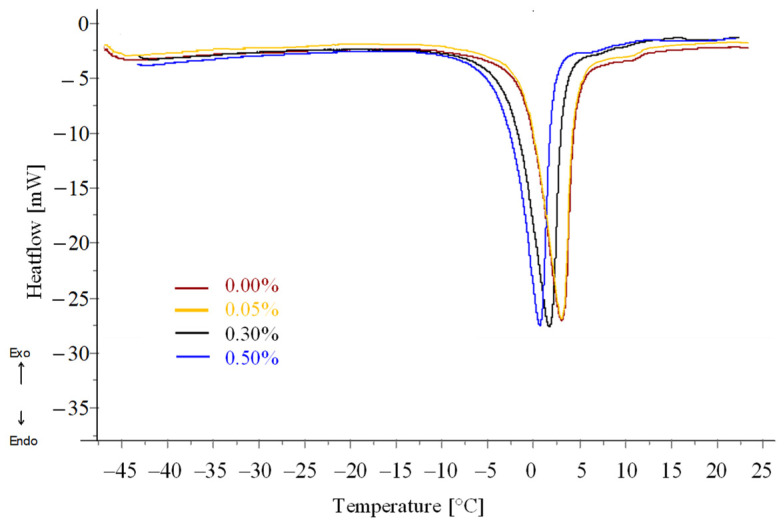
DSC melting thermograms of the control and enzyme-treated egg yolk samples, *n* = 3.

The thermophysical properties regarding melting of the yolk samples were analyzed by DSC, and the results are presented in Figure 4 and Table 5.

The onset melting temperature (T_m,onset_) was significantly influenced by enzymatic treatment. While no significant difference was observed between the control (−3.15 ± 0.34 °C) and the sample treated with 0.05% enzyme (−3.27 ± 0.42 °C), higher enzyme concentrations resulted in a marked decrease in T_m,onset_, indicating a concentration-dependent reduction in thermal stability.

This shift suggests that enzymatic hydrolysis modifies the structural organization of yolk components, likely weakening protein–lipid interactions and altering the water-binding network. In contrast, unfreezable water content (UFW) showed only a slight, non-significant increase with enzyme addition (11.67 ± 2.49% to 16.35 ± 3.00%). Although these differences were not statistically significant, the observed trend suggests enhanced water-binding capacity of enzyme-treated systems. This behavior can be attributed to structural modifications induced by enzymatic hydrolysis, which increase the availability of hydrophilic groups capable of interacting with water molecules [54,55].

## 3. Conclusions

Freezing is one of the most widely applied preservation technologies. It is efficient and economical; however, it may detrimentally influence certain properties of the products. In the case of egg yolk, freeze–thaw processing contributes to undesired changes of texture by inducing pronounced gelation. This phenomenon is characterized by increased viscosity, aggregation, and structural alterations.

An enzyme preparation with exopeptidase activity was proven to be effective in preventing freezing-induced gelation. Among the applied enzyme concentrations, 0.30 *w*/*w*% was the most effective in preserving the rheological properties of fresh, untreated control samples after freezing and thawing.

The results indicate that enzymatic hydrolysis does not prevent protein denaturation during freezing, but limits the formation of a continuous gel network. These findings were consistently supported by turbidity, rheological and DSC analyses executed during the experiment.

Overall, it can be stated that enzymatic treatment represents a promising strategy to improve the freeze–thaw stability of egg yolk, offering a viable alternative to conventional additives and supporting its broader application in frozen food systems.

For further study, it is intended to investigate the objective and subjective attributes of bakery products and different sauces, containing enzyme-treated frozen-thawed egg yolk.

## 4. Materials and Methods

### 4.1. Materials

The liquid egg yolk samples used in this study were provided by Capriovus Ltd. (Szigetcsép, Hungary). The products were produced from fresh Grade “A” hen eggs originating from caged housing systems. At the processing facility, the eggs were disinfected, broken, and separated into yolk and albumen fractions. The egg yolk was homogenized and pasteurized at 65 °C for 10 min at a mass flow rate of 600 kg/h. The final products were packaged in 1 L polyethylene terephthalate (PET) bottles, stored at 0–4 °C, and transported to the laboratory of the Hungarian University of Agriculture and Life Sciences within one day after production.

An enzyme preparation with exopeptidase activity (Flavorpro^®^ 750MDP, Biocatalysts Ltd., Cardiff, UK, 55 U/g (casein protease activity, according to manufacturer)), stored at 0–4 °C, was applied for enzymatic treatment.

Analytical-grade sodium chloride (NaCl) (Lachner, Neratovice, Czech Republic) was used for turbidity measurements.

### 4.2. Experimental Design

A total of 2 kg batches of samples were prepared for each experimental condition. The required amount of the dry enzyme preparation (Flavorpro^®^ 750MDP, Biocatalysts, Cardiff, UK) was accurately weighed and dissolved in 2 mL of distilled water in all cases to ensure a constant dilution volume across treatments. The resulting enzyme solutions were then added to the egg yolk samples to obtain final concentrations of 0.05, 0.3, and 0.5% (*w*/*w*, based on product mass). The samples were stirred with a magnetic stirrer (MS-H280 Pro, DragonLab, Beijing, China) at 300 RPM for 5 min at room temperature for homogeneity. Homogenized samples were filled into PA-PE (polyamide-polyethylene) bags (90 μm: 20 μm PA + 70 μm PE; AMCO Ltd., Budapest, Hungary) and sealed with an impulse sealer.

Enzymatic treatment was performed in a water bath at 40 °C for 120 min. Following incubation, the samples were cooled to 4 ± 1 °C. Half of the samples were subjected to freezing at −24 ± 1 °C and stored for 60 d, while the other half was analyzed immediately. Prior to analysis, frozen samples were thawed slowly by transferring them to a refrigerator at 4 ± 1 °C for 24 h.

Control samples without enzyme addition were prepared and treated under identical conditions, including homogenization, incubation, cooling, and freezing.

The same analyses were performed on both fresh (non-frozen) and frozen–thawed samples.

### 4.3. Analytical Methods

#### 4.3.1. pH Measurement

The pH of fresh and frozen–thawed samples was determined using a pH meter (Testo 206-pH2, Testo SE & Co. KGaA, Titisee-Neustadt, Germany) following calibration with pH 4.01 and 7.01 buffer solutions. Measurements were performed at 4 °C in triplicate.

#### 4.3.2. Color Measurement

Color parameters were measured using a Konica Minolta CR-400 chroma meter (Konica Minolta Sensing Europe B.V., Nieuwegein, The Netherlands) in the CIELAB color space (L*, a*, b*), representing lightness, redness, and yellowness, respectively, under standard illuminant D65 and with an aperture size of 10 mm. The instrument was calibrated prior to measurements using a standard white calibration plate according to the manufacturer’s instructions. The measurements were performed in five replicates.

Total color difference (ΔE*) was calculated according to Equation (1).

(1) $${\Delta E}^{*}=\sqrt{{{\Delta L}^{*}}^{2}+{{\Delta a}^{*}}^{2}+{{\Delta b}^{*}}^{2}}\;$$

The ranges of ΔE* were 0–1.0 (observer does not notice the difference), 1.0–2.0 (only experienced observer can notice the difference), 2.0–3.5 (unexperienced observer also notices the difference), 3.5–5.0 (clear difference in color is noticed), and >5.0 (observer notices two different colors), based on a former study [56].

#### 4.3.3. Turbidity Measurement

Turbidity of fresh and frozen–thawed samples was determined based on the method described by Wang et al. [31]. Samples were diluted in 10% (*w*/*v*) NaCl solution at a ratio of 1:100 (*w*/*w*). Absorbance was measured at 660 nm using a U-2900 spectrophotometer (Hitachi, Tokyo, Japan) in 1 cm cuvettes, with a 10% NaCl solution as a blank, as this wavelength minimizes interference from protein absorption and primarily reflects light scattering caused by particle aggregation. Six parallel measurements were performed for each sample.

#### 4.3.4. Rheological Measurements

The rheological properties were determined using an MCR 92 rotational rheometer (Anton Paar GmbH, Graz, Austria) equipped with a concentric cylinder measuring system. Measurements were conducted at 20 °C.

A shear rate sweep was applied in the range of 10–1000 s^−1^ with logarithmic increase and decrease. The shear stress and apparent viscosity were recorded every 3 s. Measurements were performed in triplicate.

Flow curves (shear stress versus shear rate) and viscosity curves (apparent viscosity versus shear rate) were assessed. Flow curves of the descending phase were fitted to the Herschel–Bulkley model (Equation (2)) using the least squares method, since this model was found adequate to describe the rheological behavior of egg yolk by several authors [45,57]. The goodness of fit was assessed by the coefficient of determination (R^2^), which exceeded 0.99 in all cases.

(2) $$\tau =\tau_{0}+K{\dot{\gamma }}^{n}$$

where τ indicates the shear stress (Pa), τ_0_ is the yield stress (Pa), $\dot{\gamma }$ refers to the shear rate (s^−1^), K is the consistency coefficient (Pa·s^n^) and n refers to the flow behavior index (dimensionless).

#### 4.3.5. Differential Scanning Calorimetry (DSC) for Determination of Protein Denaturation

Thermal denaturation of proteins was analyzed using a Micro DSC III calorimeter (Setaram, Caluire, France). Approximately 210 ± 5 mg of sample was placed in aluminum pans, with distilled water as reference.

Samples were heated from 20 °C to 95 °C at a rate of 1.5 °C/min, followed by cooling to 20 °C at 3.0 °C/min. The heat flow was recorded as a function of temperature. Denaturation enthalpy (ΔH_den_, J/g) was determined from the area under the curve, and denaturation temperature (T_den_, °C) was defined as the peak temperature. Measurements were performed in triplicate.

#### 4.3.6. Differential Scanning Calorimetry (DSC) for Determination of Melting Properties

Thermophysical properties related to melting were determined using a DSC 131 evo calorimeter (Setaram, Caluire and Montpelard, France). Samples (25–30 mg) were sealed in 100 μL aluminum pans, with an empty sealed pan used as reference.

Samples were cooled from 30 °C to −50 °C at a rate of 5.0 °C/min within the DSC instrument using a controlled cooling program, held for 15 min, and then heated to 30 °C at 2.0 °C/min. The relatively rapid cooling rate was applied to ensure well-defined freezing conditions and to minimize variability in ice crystal formation prior to the melting scan, as the evaluated parameters were determined from the subsequent heating phase. Melting enthalpy (ΔH_m_, J/g) and onset melting temperature (T_m, onset_ °C) were determined from the thermograms. ΔH_m_ was calculated from the area under the melting peak after tangential sigmoidal baseline correction, while T_m, onset_ was determined as the intersection of the baseline and the leading edge of the endothermic peak.

The unfreezable water content was calculated based on the melting enthalpy using Equation (3). Measurements were performed in triplicate.

(3) $$UFW=100-\left[\frac{{\Delta H}_{m}}{{\Delta H}_{dw}\cdot \frac{W}{100}}\cdot 100\;\right]$$

where UFW is the unfreezable water content (g/100 g), W is the moisture content of the sample (g/100 g), ΔH_m_ is the melting enthalpy of the sample (J/g), and ΔH_dw_ is the melting enthalpy of distilled water (J/g) [58].

The dry matter (DM) content of the samples was determined by oven drying at 105 ± 2 °C to constant weight. Moisture content (W) was subsequently calculated by subtracting the DM content from 100%.

### 4.4. Statistical Analysis

Statistical analysis was carried out using IBM SPSS Statistics 24 software (IBM Corp., Armonk, NY, USA) at a significance level of *p* < 0.05. Normality of residuals was assessed using the Shapiro–Wilk test, and homogeneity of variances was evaluated by Levene’s test.

One-way analysis of variance (ANOVA) was applied to determine significant differences among treatments. When significant differences were found, Tukey’s HSD test was used for multiple comparisons in the case of homogeneous variances, while the Games–Howell test was applied when the assumption of homogeneity was violated.

The results are presented as mean values with standard deviations. Different lowercase letters indicate statistically significant differences between groups (*p* < 0.05).

## Figures and Tables

**Table 1:** pH values of egg yolk samples as affected by enzymatic treatment and freeze–thaw process (mean ± SD, *n* = 3).

Enzyme Concentration (*w*/*w*%)	State	pH
0.00	Fresh	6.49 ± 0.02 ^b^
0.05	Fresh	6.49 ± 0.04 ^b^
0.30	Fresh	6.45 ± 0.03 ^bc^
0.50	Fresh	6.44 ± 0.02 ^bc^
0.00	Frozen-thawed	6.62 ± 0.02 ^a^
0.05	Frozen-thawed	6.41 ± 0.01 ^c^
0.30	Frozen-thawed	6.31 ± 0.01 ^d^
0.50	Frozen-thawed	6.32 ± 0.01 ^d^

^a–d^ Different superscript letters indicate significant differences within the column (*p* < 0.05).

**Table 2:** Color difference (ΔE) between fresh and frozen–thawed egg yolk samples as affected by enzymatic treatment.

Enzyme Concentration (*w*/*w*%)	ΔE* (Fresh vs. Frozen-Thawed)	Color Difference Interpretation *
0.00	10.37	Observer notices two different colors
0.05	9.86	Observer notices two different colors
0.30	28.23	Observer notices two different colors
0.50	29.23	Observer notices two different colors

* ΔE* values were interpreted as follows: 0–1.0 (observer does not notice the difference), 1.0–2.0 (only experienced observer can notice the difference), 2.0–3.5 (unexperienced observer also notices the difference), 3.5–5.0 (clear difference in color is noticed), and >5.0 (observer notices two different colors).

**Table 3:** Rheological parameters (τ_0_: yield stress; K: consistency coefficient; n: flow behavior index) of egg yolk samples as affected by enzymatic treatment and freeze–thaw process, based on the Herschel–Bulkley model (mean ± SD, *n* = 3).

Enzyme Concentration (*w*/*w*%)	State	τ_0_ (Pa)	K (Pa·s^n^)	n (–)
0.00	Fresh	0.00 ± 0.00 ^e^	0.14 ± 0.003 ^e^	0.92 ± 0.0004 ^d^
0.05	Fresh	0.12 ± 0.01 ^c^	0.08 ± 0.002 ^f^	0.97 ± 0.0002 ^c^
0.30	Fresh	0.10 ± 0.01 ^c^	0.07 ± 0.002 ^f^	0.99 ± 0.0002 ^a^
0.50	Fresh	0.03 ± 0.01 ^d^	0.09 ± 0.003 ^f^	0.99 ± 0.003 ^b^
0.00	Frozen-thawed	51.10 ± 0.02 ^a^	11.50 ± 0.022 ^a^	0.53 ± 0.0029 ^h^
0.05	Frozen-thawed	0.76 ± 0.01 ^b^	1.09 ± 0.012 ^b^	0.74 ± 0.0001 ^g^
0.30	Frozen-thawed	0.00 ± 0.00 ^e^	0.22 ± 0.008 ^d^	0.87 ± 0.00001 ^e^
0.50	Frozen-thawed	0.02 ± 0.02 ^de^	0.40 ± 0.011 ^c^	0.84 ± 0.0004 ^f^

^a–h^ Different superscript letters indicate significant differences within the column (*p* < 0.05).

**Table 4:** Denaturation enthalpy (ΔH_d_) and denaturation temperature (T_d_) of egg yolk samples as affected by enzymatic treatment and freeze–thaw process, determined by DSC (mean ± SD, *n* = 3).

Enzyme Concentration (*w*/*w*%)	State	ΔH_d_ (J/g)	T_d_ (°C)
0.00	Fresh	1.19 ± 0.03 ^a^	76.60 ± 0.42 ^c^
0.05	Fresh	1.15 ± 0.04 ^ab^	77.42 ± 0.23 ^bc^
0.30	Fresh	1.08 ± 0.02 ^b^	78.51 ± 0.31 ^ab^
0.50	Fresh	1.09 ± 0.02 ^b^	78.62 ± 0.05 ^a^
0.00	Frozen-thawed	0.84 ± 0.02 ^c^	76.67 ± 0.54 ^c^
0.05	Frozen-thawed	0.87 ± 0.01 ^c^	77.73 ± 0.19 ^b^
0.30	Frozen-thawed	0.87 ± 0.03 ^c^	78.52 ± 0.20 ^ab^
0.50	Frozen-thawed	0.88 ± 0.02 ^c^	78.58 ± 0.03 ^a^

^a–c^ Different superscript letters indicate significant differences within the column (*p* < 0.05).

**Table 5:** Thermal properties (T_m, onset_: onset melting temperature; UFW: unfreezable water content) of egg yolk samples as affected by enzymatic treatment, determined by DSC (mean ± SD, *n* = 3).

Enzyme Concentration (*w*/*w*%)	T_m, onset_ (°C)	UFW (%)
0.00	−3.15 ± 0.34 ^a^	11.67 ± 2.49 ^a^
0.05	−3.27 ± 0.42 ^a^	11.17 ± 1.57 ^a^
0.30	−5.42 ± 0.33 ^b^	15.00 ± 1.63 ^a^
0.50	−6.26 ± 0.20 ^b^	16.35 ± 3.00 ^a^

^a,b^ Different superscript letters indicate significant differences within the column (*p* < 0.05).

## Data Availability

The data used in this study are available upon request.
